# Electrochemistry of Carbon Materials: Progress in Raman Spectroscopy, Optical Absorption Spectroscopy, and Applications

**DOI:** 10.3390/nano13040640

**Published:** 2023-02-06

**Authors:** Marianna V. Kharlamova, Christian Kramberger

**Affiliations:** 1 Centre for Advanced Materials Application (CEMEA) of Slovak Academy of Sciences, Dúbravská cesta 5807/9, 845 11 Bratislava, Slovakia; 2 Faculty of Physics, University of Vienna, Bolzmanngasse 5, 1090 Vienna, Austria

**Keywords:** electrochemistry, carbon nanotube, spectroelectrochemistry, voltamerometry, Raman spectroscopy, optical absorption spectroscopy

## Abstract

This paper is dedicated to the discussion of applications of carbon material in electrochemistry. The paper starts with a general discussion on electrochemical doping. Then, investigations by spectroelectrochemistry are discussed. The Raman spectroscopy experiments in different electrolyte solutions are considered. This includes aqueous solutions and acetonitrile and ionic fluids. The investigation of carbon nanotubes on different substrates is considered. The optical absorption experiments in different electrolyte solutions and substrate materials are discussed. The chemical functionalization of carbon nanotubes is considered. Finally, the application of carbon materials and chemically functionalized carbon nanotubes in batteries, supercapacitors, sensors, and nanoelectronic devices is presented.

## 1. Introduction

**Figure 1:**
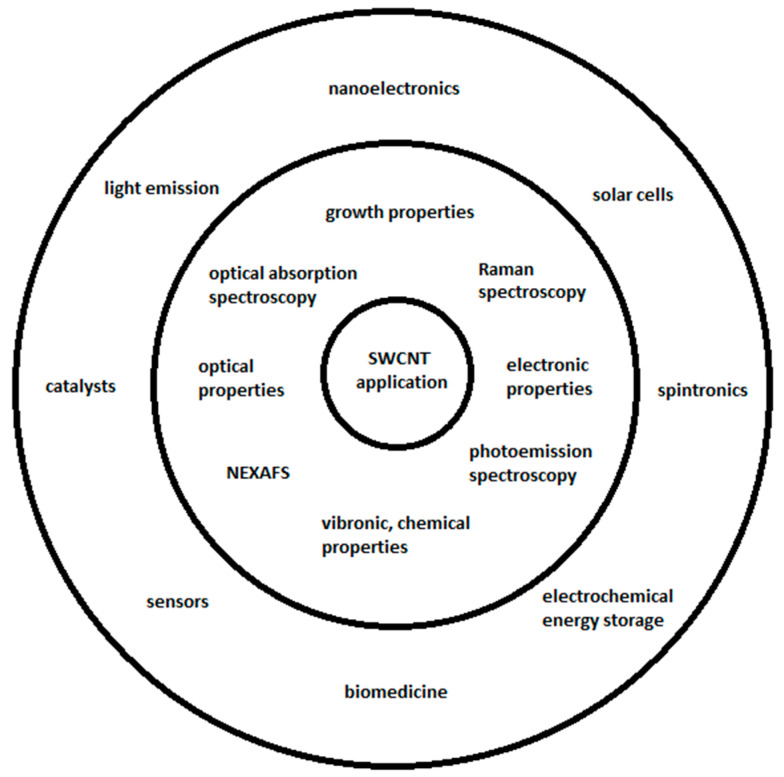
SWCNT properties, methods, and applications.

Carbon nanotubes include single-walled carbon nanotubes (SWCNTs) with unique properties [1,2] and multi-walled carbon nanotubes (MWCNTs). SWCNTs are sorted and separated to obtain uniform electronic properties [3], and the channels of SWCNTs are filled [4,5] with substances [6,7] for the investigation of kinetics and electronic properties [8,9,10,11,12,13,14,15] for applications (Figure 1). The SWCNTs are filled with inorganic compounds [16,17,18,19,20,21,22,23,24,25,26,27,28,29,30,31,32,33,34,35,36,37,38,39,40,41,42,43,44,45,46], molecules [47,48,49,50,51,52,53,54,55,56,57,58,59,60,61,62,63,64,65,66,67,68,69,70,71,72,73,74,75,76], and elementary substances [77,78,79,80,81,82,83,84,85,86,87,88,89,90,91,92,93,94,95,96,97,98,99,100,101,102,103,104,105,106,107,108]. Inorganic substances are introduced inside carbon nanotubes by the gas phase and liquid phase methods. Molecules are filled inside carbon nanotubes by the gas phase and solution methods. Elementary substances are incorporated inside carbon nanotubes by the gas, solution, and melt methods. The electronic properties of chemically modified carbon material are investigated by Raman spectroscopy, near-edge X-ray absorption fine structure spectroscopy (NEXAFS), optical absorption spectroscopy (OAS), and photoemission spectroscopy.

A high current carrying capacity, long cycling stability, excellent electrical conductance, and good capability in rapid charge and discharge make SWCNTs electrodes possible, leading to a high performance [109]. 

The electrochemical doping allows for the alteration of the doping level [110,111,112]. The shift in the Fermi level is proportional to the applied voltage [112,113]. The experimental parameters are varied to achieve perfect conditions [112].

In 1999, the first example of electrochemical doping appeared [114]. The methods that investigated the modified electronic properties are voltamperometry [115,116,117,118,119] and spectroelectrochemistry [110,113,120,121,122,123,124,125,126,127,128,129,130,131,132,133,134,135,136,137,138,139,140,141,142]. Voltamperometry allows for evaluating the reversibility of the p- and n-doping of SWCNTs by applied voltage using the cyclic voltamerograms [112]. Combining the spectroscopic investigations, such as Raman spectroscopy and optical absorption spectroscopy, with electrochemical charging allows for the evaluation of the modifications of the electronic properties of SWCNTs [143]. 

The aim of this review is to summarize the reports on the investigations of the electrochemical properties of carbon material. In Section 2, the results of the voltamperometry are discussed. In Section 3, the results of the spectroelectrochemistry with Raman spectroscopy are considered, i.e., measuring the spectra with electrochemical charging. In Section 4, the results of the spectroelectrochemistry with optical absorption spectroscopy are presented. In Section 5, investigations of chemically functionalized carbon nanotubes are highlighted. In Section 6, applications of carbon material in electrochemical devices are discussed. 

## 2. Voltamperometry

For voltamperometry, it is important that the MWCNTs do not destroy under electrochemical charging. MWCNTs are stable up to a ~±2 eV applied potential. This allows for one to use them for voltamperometry measurements. Measurements are conducted at different scan rates. The data present the dependence of the voltage on the capacity. The insertion of the electrolyte happens. For MWCNTs, a capacity of up to ~1000 mAh/g was observed. 

In Ref. [116], the electrochemical experiments were performed with lithium insertion into nanotubes. High values of irreversible capacity C_irr_ (from 460 to 1080 mAh/g) were obtained. The authors plotted the dependence of C_irr_ on the mesopore volume, which showed the linear behavior [116].

**Figure 2:**
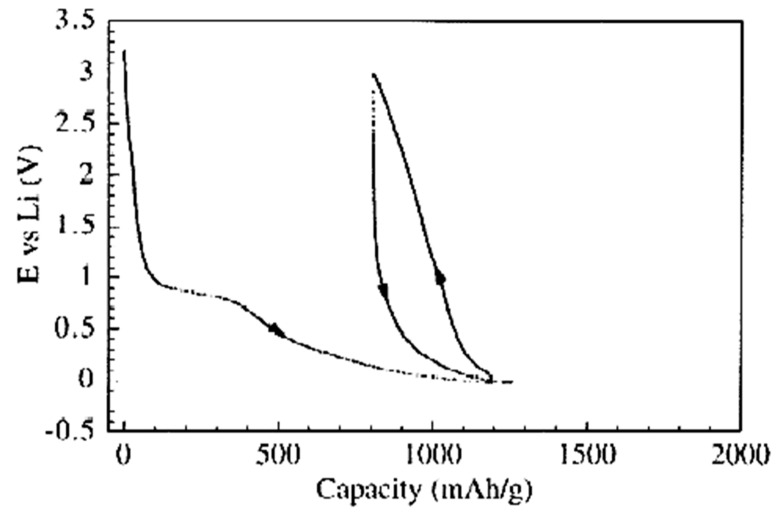
Galvanostatic insertion–extraction of lithium into nanotubes. Current load of 20 mA/g. Reprinted from Frackowiak E, Beguin F Carbon 40 1775 (2002), Copyright (2002), with permission from Elsevier [116].

Figure 2 presents the voltamperometric data of lithium insertion and extraction in MWCNTs to test electrodes from carbon tubular form. They show the typical behavior of tubular carbon [116]. The irreversible capacity values of all forms of tubular carbon C_irr_ are extremely high. It is believed that one factor that makes the value of C_irr_ is the solid electrolyte interphase (SEI) formation. 

**Figure 3:**
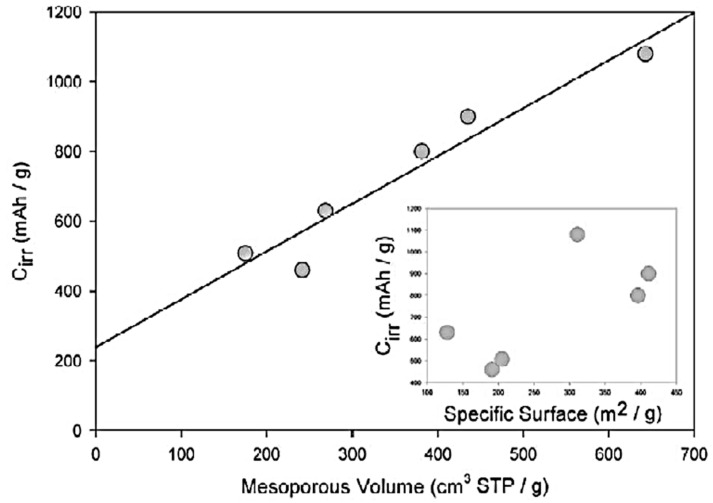
Dependence of irreversible capacity C_irr_ versus mesopore volume and specific surface area (inset) for different types of nanotubes. Reprinted from Frackowiak E, Beguin F Carbon 40 1775 (2002), Copyright (2002), with permission from Elsevier [116].

The authors of Ref. [116] plotted the C_irr_ data versus the mesopore volume. Figure 3 presents the linear dependence with the liner fitting of multi-walled carbon nanotubes, single-walled carbon nanotubes, and carbon filaments [116]. This is caused by the tubular structure of carbon forms, with an easy access of voluminous solvated lithium cations in the inner part of the electrode where it decomposes [116].

**Figure 4:**
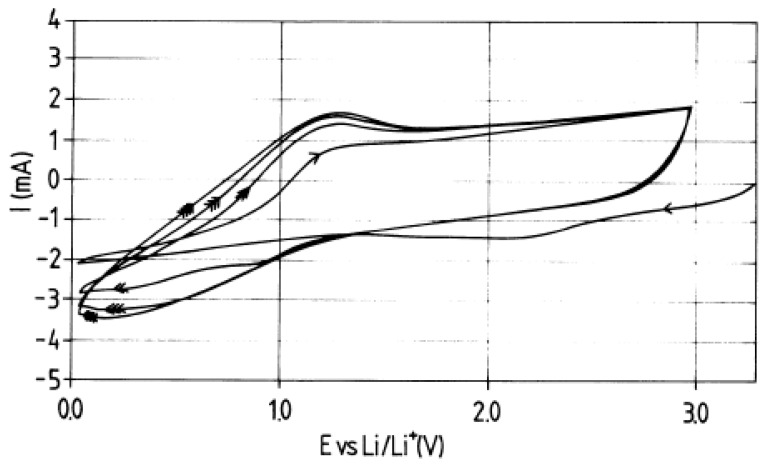
Voltammetry characteristics of MWCNT. Reprinted from E. Frackowiak et al./Carbon 37 (1999) 61–69, Copyright (1999), with permission from Elsevier [144].

In Ref. [144], the voltamperometry data of MWCNT at different scan rates are presented (Figure 4). Until 1.5 V (open-circuit voltage (OCV)) and about 1 V, there are the reductions of chemicals, and from about 0.5 V until 0 V, there is the lithium insertion. Above 1.5 V, the region of charging of the double layer was observed, which testified to the good electrochemical properties [114].

## 3. Spectroelectrochemistry with Raman Spectroscopy

The electrochemical experiments are combined with Raman spectroscopy and optical absorption spectroscopy. Yet, other methods are possible.

To present the method, in the spectroelectrochemical technique, SWCNTs serve as a working electrode. The spectrum is measured under an applied potential. The spectra obtained at different applied voltages are plotted for comparison and tracing changes.

Historically, the first experiment on the electrochemical doping of SWCNTs was performed in 1999 [142]. In this experiment, Raman spectroscopy was applied, and the electrolyte was sulfuric acid. In 2000, the experiments in aqueous [118] and aprotonic (tetrahydrofuran) [110] solutions were made. In the literature, there are also reports on experiments in aqueous [113,120,121,122,123,124,125,126,134,135,136,137,138,139,140] solutions and acetonitrile [119,127,128,130,131] and ionic fluids [129]. Thin films [118,119,121,122,128,129,130,134,135,136] and buckypaper [120,125,137,138,139,143] were analyzed.

**Figure 5:**
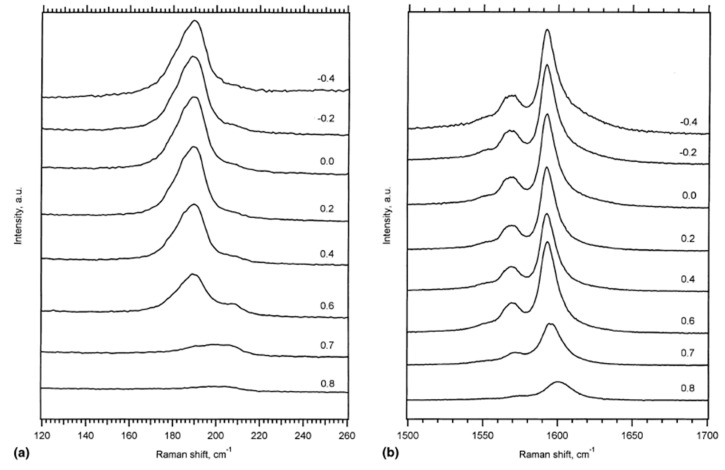
The resonance Raman spectra of the RBM (**a**) and TDM (**b**) modes acquired at a laser wavelength of 514 nm under both positive and negative voltages. Reprinted from Kavan L, Rapta P, Dunsch L Chem. Phys. Lett. 328 363 (2000), Copyright (2000), with permission from Elsevier [118].

In Ref. [118], the authors studied the Raman spectra of SWCNTs upon electrochemical charging at applied potentials from −0.2 to −0.8 eV and from +0.2 to +1.4 eV. Figure 5 shows the RBM and tangential displacement modes (TDM) of the resonance Raman spectra of a buckypaper in a 1 M NaCl aqueous solution acquired at a 514 nm laser [118].

**Figure 6:**
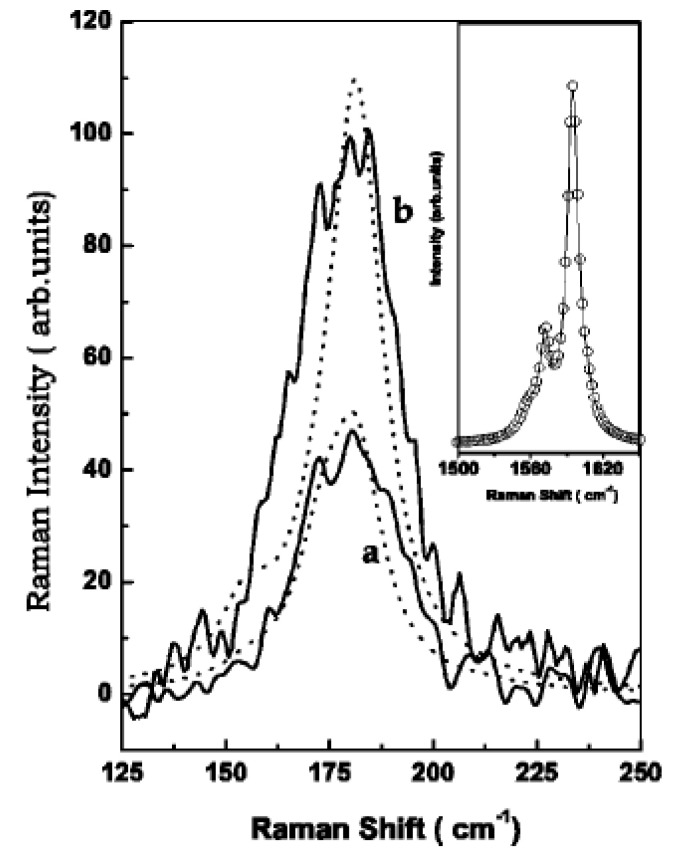
Experimental Raman spectra (solid lines) of the RBM at zero bias (**a**) and −0.08 V (**b**) for a laser energy of 2.41 eV. The dotted lines are the calculated line shapes. The inset shows the G-band at zero bias (open circles) and at −0.08 V (solid line). Reprinted from Ghosh S, Sood A K, Rao C N R J. Appl. Phys. 92 1165 (2002), with the permission of AIP Publishing [123].

The authors of Ref. [123] investigated the RBM-band of Raman spectra. In the RBM band, there is the peak at 180 cm^−1^. In the G-band, there is the peak of the tangential modes (TM) at 1590 cm^−1^. Figure 6 shows experimental Raman spectra (solid lines) of the RBM at zero bias (Figure 6a) and −0.08 V (Figure 6b) for an applied laser energy of 2.41 eV. The dotted lines are the calculated line shapes. The inset shows the G-band at zero bias (open circles) and at −0.08 V (solid line). Thus, the intensity and position of peaks change under electrochemical charging [123].

**Figure 7:**
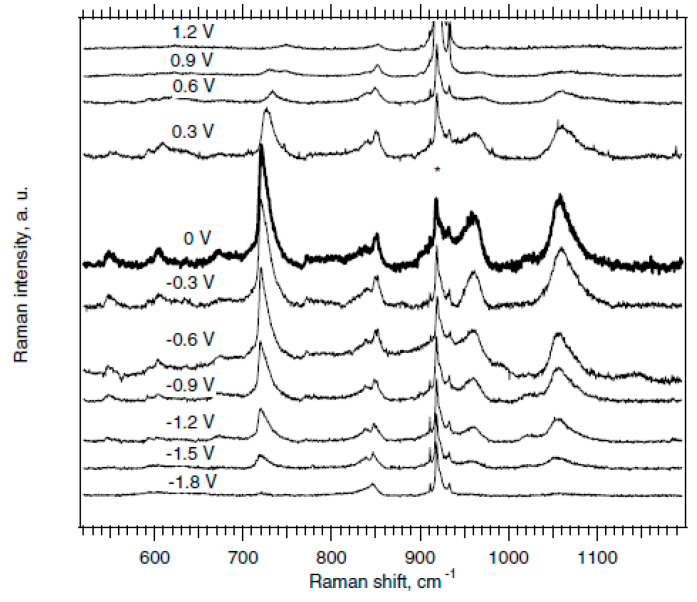
Potential dependent Raman spectra (excited at 2.18 eV) of HiPco SWCNT on the Pt electrode in acetonitrile +0.2 M LiClO_4_. The peaks marked by * are assigned to the electrolyte solution. Reprinted with permission from Ref. [127], copyright 2006 Wiley-VCH Verlag GmbH & Co. KGaA, Weinheim.

In Ref. [127], the potential dependent Raman spectra (excited at 2.18 eV) of HiPco SWCNT were obtained. There are intermediate frequency modes (IFM) in the spectrum. Figure 7 shows the dependence of IFM modes on electrochemical charging from −1.8 V to 0 V and from 0 V to 1.2 V. The dispersive IFM modes are shifted stronger than the non-dispersive IFM modes upon p-doping, whereas n-doping has the opposite effect. Thus, one laser wavelength is enough to recognize dispersive and non-dispersive features [127].

Thus, the spectroelectrochemistry technique is a modern, useful, and powerful method based on the Raman spectroscopy method. The substances for electrodes should be stable in electrolyte solutions, and they should not destroy under applied voltages. Raman maps are plotted using the obtained data; they are the dependence of the Raman peak position on an applied voltage. In Raman maps of carbon nanotubes, the modifications of the electronic properties are observed.

## 4. Spectroelectrochemistry with Optical Absorption Spectroscopy

The optical absorption spectroscopy was first combined with electrochemical charging in the year 2000 [118]. Indium-tin oxide (ITO) [118,119,124,125] and think Pt film [124,125] were used as substrates. Aqueous [118], acetonitrile [118,119,124,125], and ionic liquids [125] were used. 

**Figure 8:**
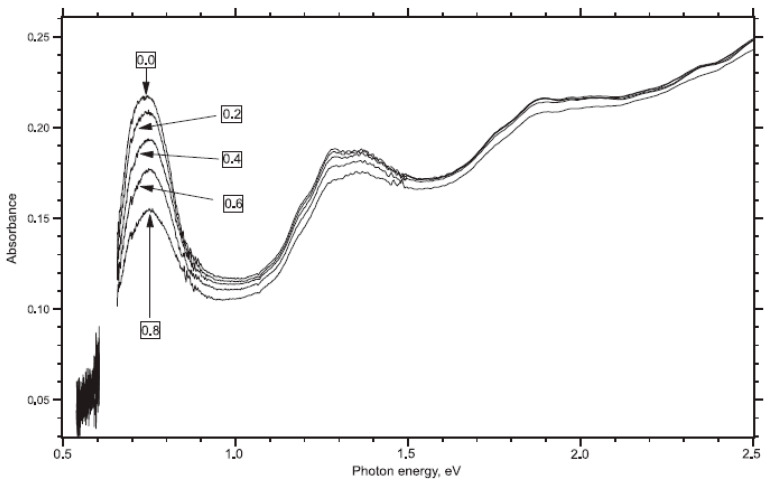
Vis-NIR spectra of ITO-supported SWCNT in aqueous 0.1M KCl saturated with nitrogen. Reprinted from Kavan L, Rapta P, Dunsch L Chem. Phys. Lett. 328 363 (2000), Copyright (2000), with permission from Elsevier [118].

In Ref. [118], electrochemical experiments were conducted using the ITO substrate and aqueous 0.1 M KCl saturated with nitrogen as the electrolyte solution. Figure 8 shows the OAS spectra of SWCNTs measured with applied potentials from 0 V to 0.8 V [118]. The spectra contain absorption bands at 1800 nm (0.68 eV), 1000 nm (1.3 eV), and 700 nm (1.9 eV). Upon electrochemical charging, there is the suppression of the absorption bands. This was explained by a shift in the Fermi level.

**Figure 9:**
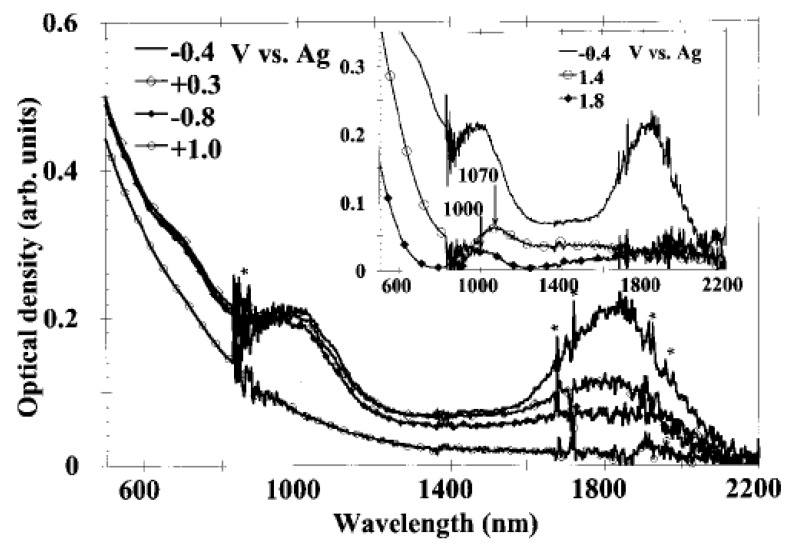
In situ absorption spectra of an SWCNT film. The star indicates features coming from the solvent and also noises. Reprinted from Kazaoui S et al. Appl. Phys. Lett. 78 3433 (2001), with the permission of AIP Publishing [133].

In Ref. [133], the in situ measurement of SWCNT films under electrochemical charging was performed. Figure 9 shows the OAS spectra at constant electrode potentials [133]. The potentials of −0.4 V, −0.8 V, 0.3 V, 1.0 V, 1.4 V, and 1.8 V were applied. Upon the application of the potential, the absorption bands at 1800 nm, 1000 nm, and 700 nm disappear. At high positive potentials, new broad peaks appear around 1070 nm (1.15 eV) at 1.4 V and around 1000 nm (1.24 eV) at 1.8 V. At low negative potentials, no new absorption bands, at least up to 21.4 V, were observed. At high positive potentials, the authors also observed the increased absorption background in the near-infrared field [133]. 

## 5. Investigations of Chemically Functionalized Carbon Nanotubes

There is a large field of work on chemically functionalized carbon nanotubes. It was shown that metal halogenide-filled SWCNTs with a decreased [145,146,147,148,149,150,151,152,153,154,155,156,157,158,159,160] Fermi level, metal chalcogenide-filled SWCNTs [161,162,163], metal-filled SWCNTs [164,165,166], metal halogenide-filled SWCNTs [167], and metallocene-filled SWCNTs [168,169,170,171,172,173,174,175,176,177,178,179,180,181] with an increased Fermi level can be used for the electrochemical measurements. 

### 5.1. Covalent Functionalization of Carbon Nanotubes

The covalent functionalization of carbon nanotubes was made with fluorination. This method is performed with different chemicals and synthesis parameters [182,183,184,185,186]. 

**Figure 10:**
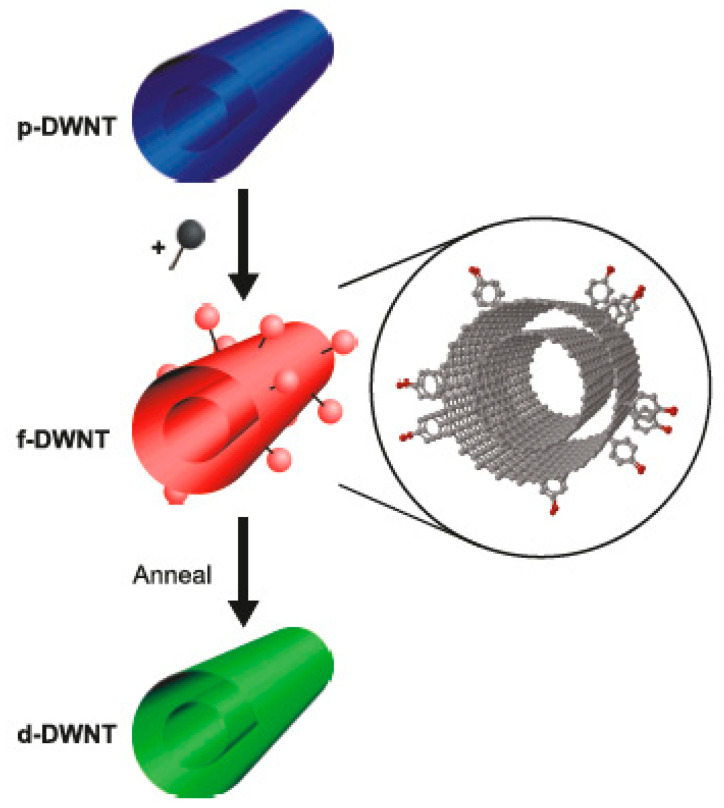
The schematics of functionalization and reversible defunctionalization by the thermal treatment of DWCNTs. Reprinted with permission from Bouilly D et al. ACS Nano 2011 5 6 4927. Copyright 2011 American Chemical Society [182].

In Ref. [182], the covalent functionalization of double-walled carbon nanotubes (DWCNTs) with aryldiazonium salt was performed, and it was shown that the functionalization is reversible upon thermal treatment. The DWCNT transistors were constructed based on the functionalized DWCNTs, and the assignment of the metallicity of the inner and outer walls of DWCNTs was conducted (Figure 10). 

**Figure 11:**
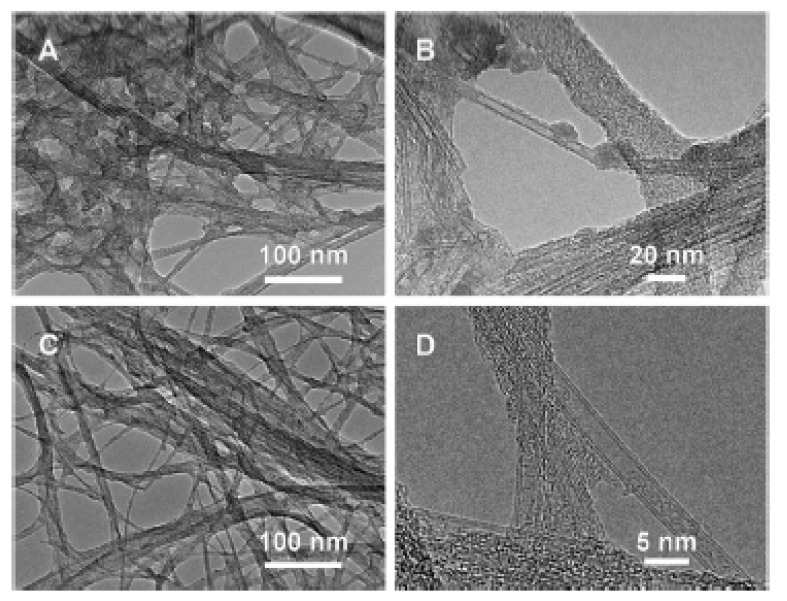
The TEM data in low- and high-magnification images of DWCNTs treated with 5 mL of a solution for 24 h (**A**,**B**) and treated with 10 mL of a solution for 2 h (**C**,**D**). Reprinted with permission from Brozena A et al. JACS 2010 132 11 3932. Copyright 2010 American Chemical Society [183].

In Ref. [183], the outer walls of SWCNTs were selectively oxidized by oleum and nitric acid. Figure 11 shows the transmission electron microscopy (TEM) data of oxidized nanotubes. In Figure 11A,B low- and high-magnification images of DWCNTs treated with 5 mL of a solution for 24 h are shown. In Figure 11C,D DWCNTs treated with 10 mL of a solution for 2 h are presented [183].

**Figure 12:**
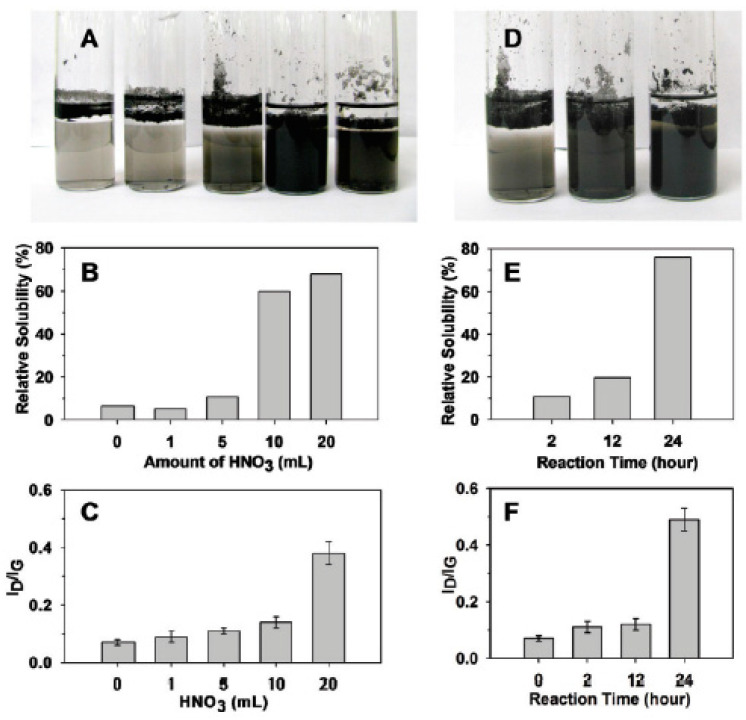
The solubility and defectiveness of DWCNTs at a fixed reaction time of 2 h with nitric acid (**A**–**C**) and increasing reaction times using 5 mL of HNO_3_ (**D**–**F**). Reprinted with permission from Brozena A et al. JACS 2010 132 11 3932. Copyright 2010 American Chemical Society [183].

In Ref. [183], the samples were further investigated at a fixed reaction time of 2 h with nitric acid (Figure 12A–C) and an increasing reaction time using 5 mL of HNO_3_ (Figure 12D–F). In Figure 12B, it is seen that the relative solubility of DWCNTs is increased by increasing the amount of acid. In Figure 12C, it is visible that the nanotubes become more defective, because the ratio of the peaks in the Raman spectra of I_D_/I_G_ is increased. By increasing the reaction time (Figure 12D–F), the solubility and defectiveness of nanotubes increase, too. This is caused by the appearance of more carboxylic groups on the surface of DWCNTs with oxidation. This leads to more solubility and more defects [183].

**Figure 13:**
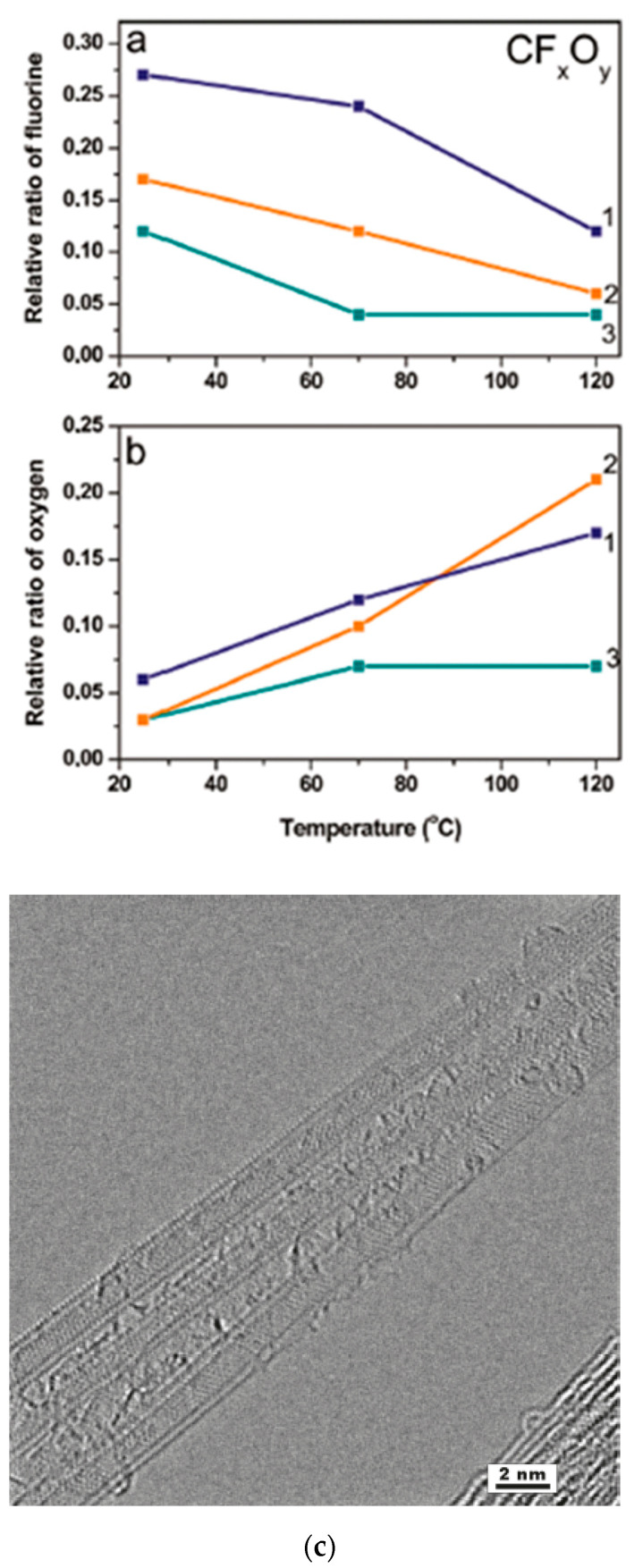
Concentration of fluorine (**a**) and oxygen (**b**) in the DWCNTs fluorinated by F_2_ (1), BrF_3_ (2), and CF_4_ plasma (3) as a function of the annealing temperature. (**c**) High-resolution TEM image of DWCNTs fluorinated by F_2_ at 200 °C. Reprinted with permission from Bulusheva L et al. Chem Mater 2010 22 4197. Copyright 2010 American Chemical Society [184].

In Ref. [184], DWCNTs were fluorinated using (1) gaseous F_2_ at 200 °C, (2) a mixture of BrF_3_ and Br_2_ at room temperature, and (3) radio frequency CF_4_ plasma. Figure 13a shows the relative ratio of fluorine plotted versus the synthesis temperature. Figure 13b shows the relative ratio of oxygen plotted versus the experiment temperature. Figure 13c demonstrates the high-resolution TEM image of DWCNTs fluorinated by F_2_ at 200 °C [184].

**Figure 14:**
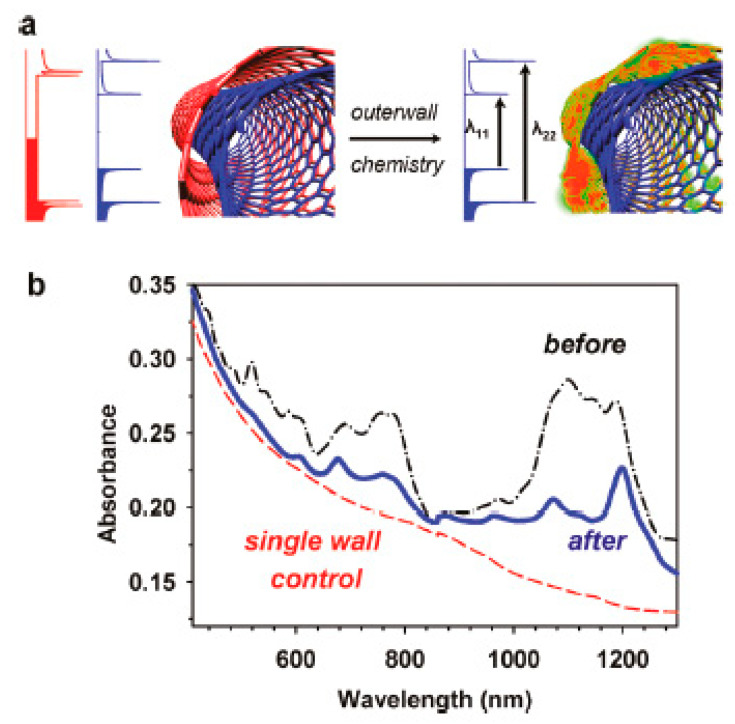
(**a**) The schematics of the covalent functionalization of DWCNTs. (**b**) The optical absorption spectrum of DWCNTs before and after the functionalization with diazonium salts. Reprinted with permission from Piao Y. et al. J Phys Chem Lett 2011 2 1577. Copyright 2011 American Chemical Society [185].

In Ref. [185], the covalent modification of DWCNTs was performed to control the sidewall chemistry. Figure 14a shows the schematics of the covalent functionalization. Figure 14b demonstrates the optical absorption spectrum of DWCNTs before and after the functionalization with diazonium salts. It is visible that the covalent functionalization leads to a charge transfer in DWCNTs [185]. 

### 5.2. Gas Sorption on Carbon Nanotubes

Gas sorption on carbon nanotubes is possible. It can be reversible depending on the experimental conditions [187,188]. This allows for using carbon nanotubes as gas sensors, biosensors, and sensors for liquids. The reversibility of adsorption is the main characteristic for the implementation in sensors.

### 5.3. Substitution of Carbon Atoms with Other Atoms

Many studies were dedicated to the substitution of carbon atoms of carbon nanotubes by other atoms. The electronic properties depend on the type and concentration of the doping atoms [189]. The first experiment was made in 1993 [190], and after that, many examples of doping with nitrogen [191,192,193,194,195,196,197,198,199,200,201] and boron [202,203,204,205,206,207,208,209] were made.

**Figure 15:**
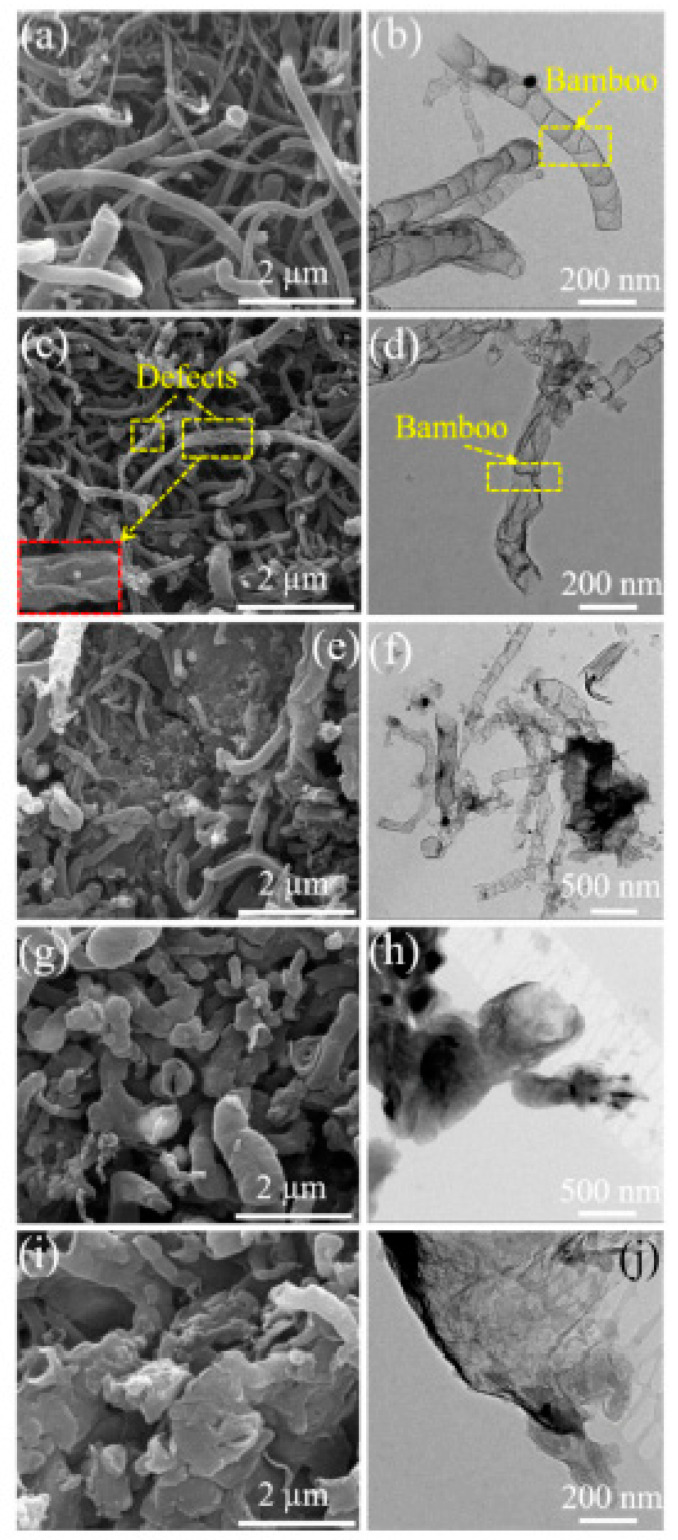
The SEM and TEM images of B,N-CNTs at different addition amounts of boric acid (B,N-CNT-1, 0.05 g (**a**,**b**), B,N-CNT-2, 0.1 g (**c**,**d**), B,N-CNT-3, 0.25 g (**e**,**f**), B,N-CNT-4, 0.5 g (**g**,**h**), and B,N-CNT-5, 1 g (**i**,**j**)). Copyright 2021 by the authors. Licensee MDPI, Basel, Switzerland. This article is an open-access article distributed under the terms and conditions of the Creative Commons Attribution (CC BY) license [210].

In Ref. [210], nitrogen-doped carbon nanotubes were boron-doped. Figure 15 shows the SEM and TEM images of B,N-CNTs at different addition amounts of boric acid (B,N-CNT-1, 0.05 g, B,N-CNT-2, 0.1 g, B,N-CNT-3, 0.25 g, B,N-CNT-4, 0.5 g, and B,N-CNT-5, 1 g). These images are amazing examples of the unique chemistry of carbon nanotubes. 

**Figure 16:**
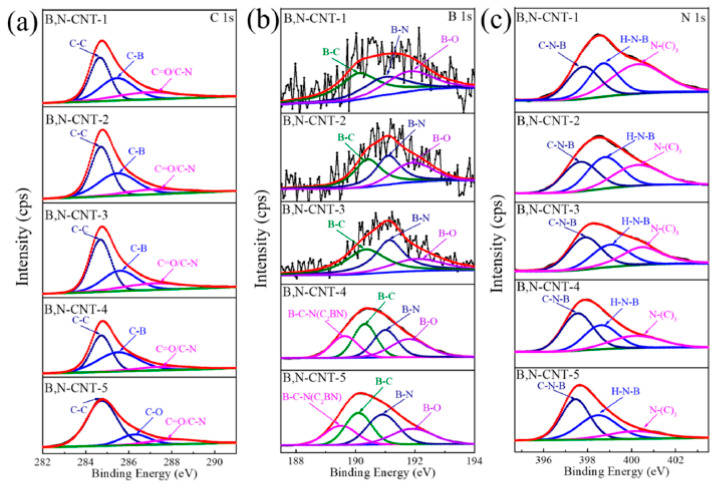
The (**a**) C 1s XPS spectrum, (**b**) B 1s XPS spectrum, and (**c**) N 1s XPS spectrum of B,N-CNTs (B,N-CNT-1, 0.05 g, B,N-CNT-2, 0.1 g, B,N-CNT-3, 0.25 g, B,N-CNT-4, 0.5 g, and B,N-CNT-5, 1 g). Copyright 2021 by the authors. Licensee MDPI, Basel, Switzerland. This article is an open-access article distributed under the terms and conditions of the Creative Commons Attribution (CC BY) license [210].

In Figure 16, the surface chemical compositions of B,N-CNTs calculations with XPS are shown. Figure 16a shows the C 1s XPS spectra of samples. Figure 16b shows the B1s XPS spectra of samples. Figure 16c shows the N 1s XPS spectra of the samples [210]. 

### 5.4. Intercalation of Nanotube Bundles

The intercalation of carbon nanotube bundles means incorporating simple substances and chemical compounds to the space between nanotubes in the bundles. The intercalation with p- and n-dopants was demonstrated. The electronic properties of SWCNTs were investigated by Raman spectroscopy, optical absorption spectroscopy, electron energy loss spectroscopy, and X-ray photoelectron spectroscopy techniques [211,212,213,214,215,216,217,218,219,220,221,222,223,224,225,226,227,228,229,230,231,232,233,234,235,236,237].

### 5.5. Filling of Carbon Nanotubes

**Figure 17:**
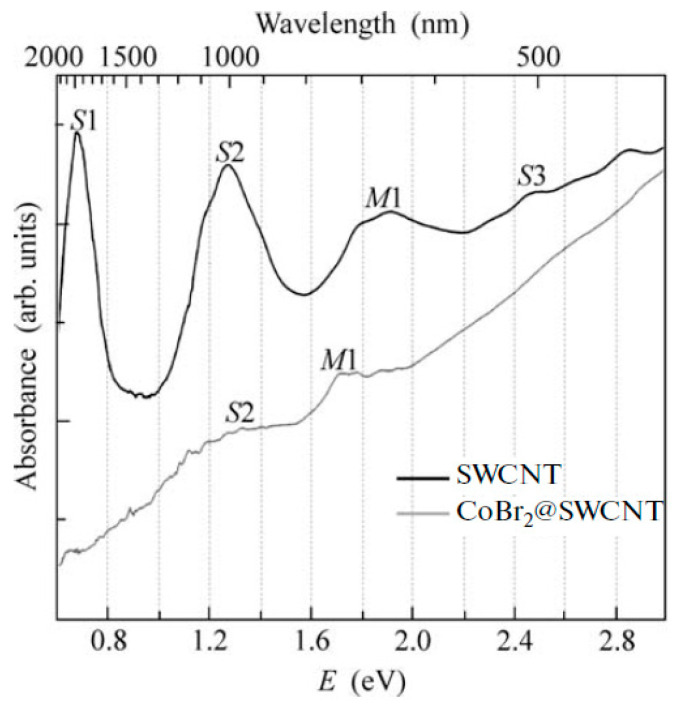
The OAS spectra of pristine and cobalt bromide-filled SWCNTs. Reprinted from M.V. Kharlamova et al. Study of the electronic structure of single-walled carbon nanotubes filled with cobalt bromide, JETP Letters, V. 91, n 4, p. 196–200, 2010, Springer Nature [146].

OAS spectroscopy is used to identify the charge transfer in filled SWCNTs [145,146,147,148,149,150,151,152,153,154,155,156,157,158,159,160,161,162,163,164,165,166,167,168,169,170,171,172,173,174,175,176,177,178,179,180,181]. Figure 17 shows the OAS spectra of pristine and cobalt bromide-filled SWCNTs [146]. The modification of spectra such as the change in the intensity of peaks, the shift of peaks, the alteration of peak profiles, and the disappearance and appearance of peaks testifies to the charge transfer in filled SWCNTs.

**Figure 18:**
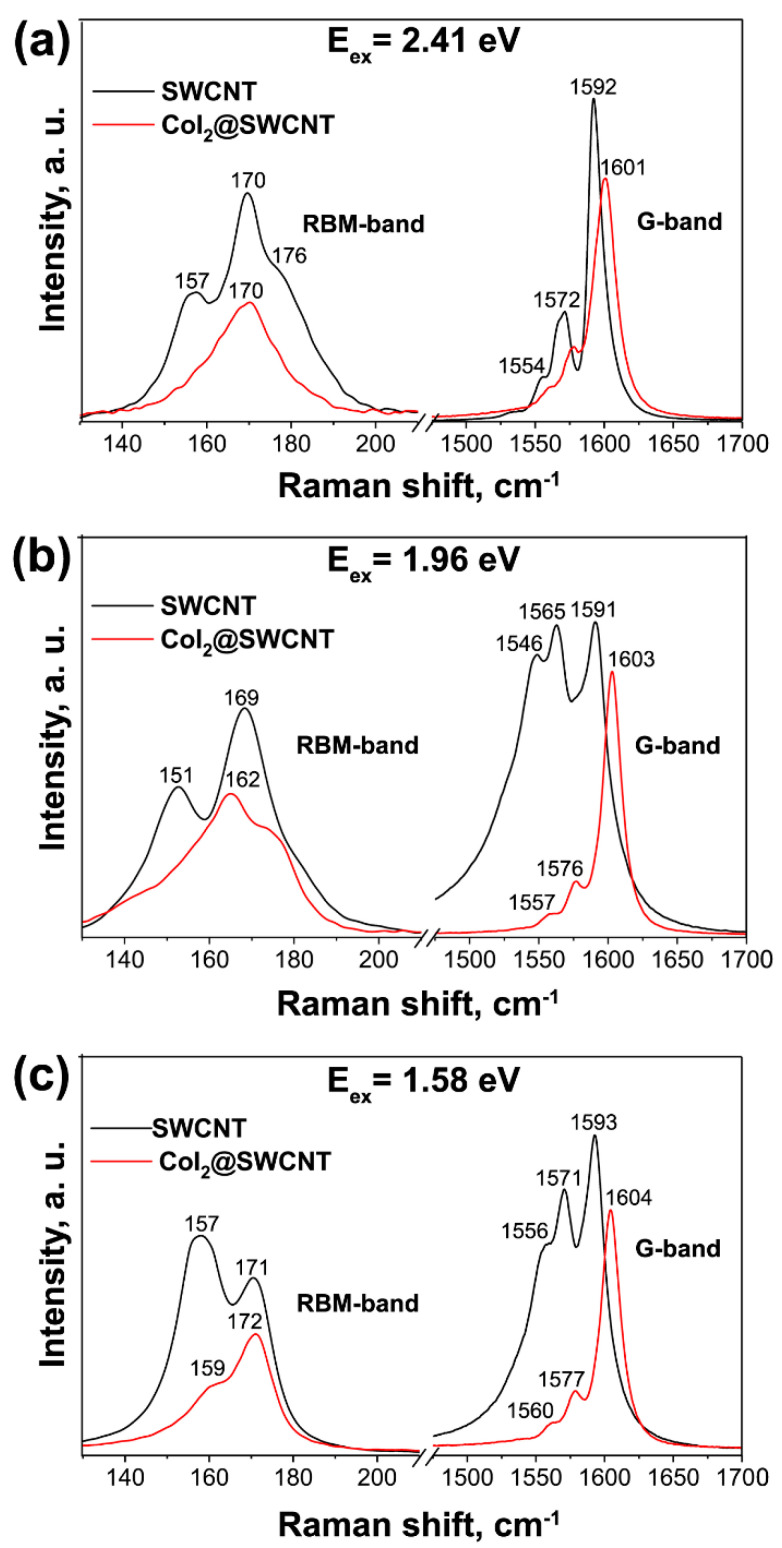
The Raman spectra of pristine and cobalt iodide-filled SWCNTs obtained at laser energies of 2.41 eV (**a**), 1.96 eV (**b**), 1.58 eV (**c**). Copyright 2022 by the author. Licensee MDPI, Basel, Switzerland. This article is an open-access article distributed under the terms and conditions of the Creative Commons Attribution (CC BY) license [238].

Raman spectroscopy is used to identify the charge transfer and electronic and vibronic properties of filled SWCNTs [181,182,183,184,185]. Figure 18 shows the Raman spectra of pristine and cobalt iodide-filled SWCNTs obtained at different laser wavelengths [238]. The modification of spectra such as the change in the intensity of peaks, the shift of peaks, and the alteration of peak profiles testifies to the charge transfer in filled SWCNTs.

**Figure 19:**
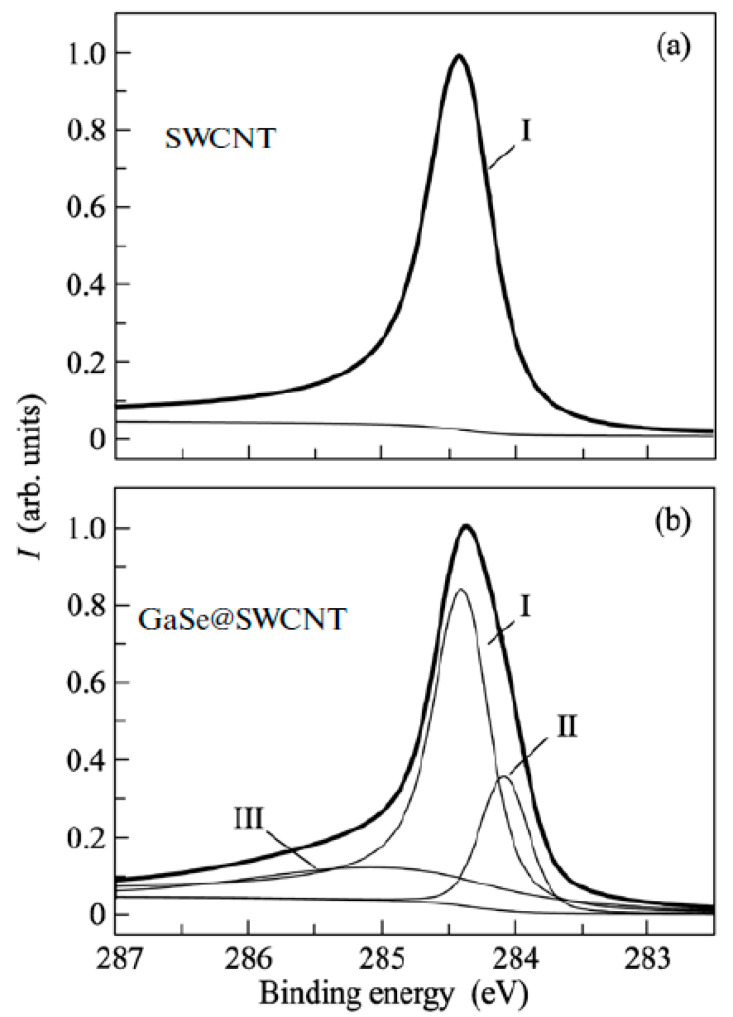
The XPS spectra of pristine (**a**) and gallium selenide-filled SWCNTs (**b**). Reprinted from M.V. Kharlamova. Novel approach to tailoring the electronic properties of single-walled carbon nanotubes by the encapsulation of high-melting gallium selenide using a single-step process, JETP letters, V. 98, n 5, p. 272–277, 2013, Springer Nature [162].

XPS is used to identify the direction and value of the Fermi level shift of the filled SWCNTs [145,146,147,148,149,150,151,152,153,154,155,156,157,158,159,160,161,162,163,164,165,166,167,168,169,170,171,172,173,174,175,176,177,178,179,180,181]. Figure 19 shows the XPS spectra of pristine and gallium selenide-filled SWCNTs [162]. The C 1s XPS spectra showed the shift of the peak and the change in its width. UPS was also used as a direct method of the investigation of the Fermi level shift in filled SWCNTs [145,146,147,148,149,150,151,152,153,154,155,156,157,158,159,160,161,162,163,164,165,166,167,168,169,170,171,172,173,174,175,176,177,178,179,180,181].

**Figure 20:**
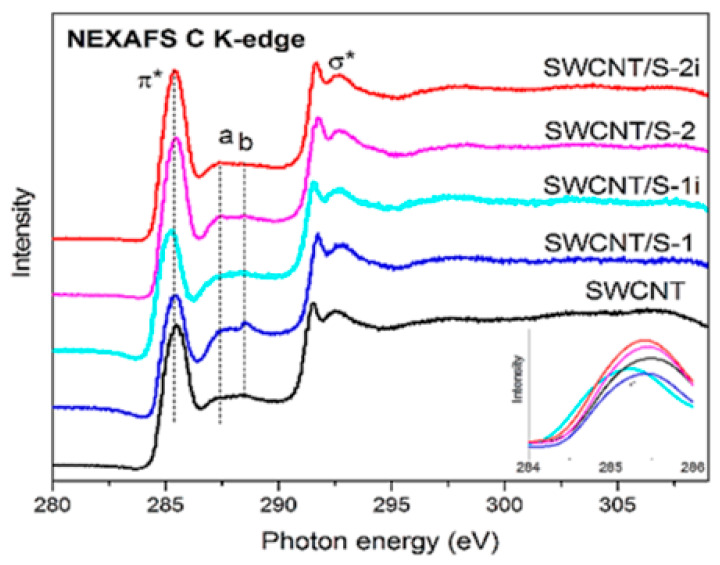
The NEXAFS spectra of the pristine and sulfur-filled SWCNTs and the processed samples. Copyright 2020 by the author. Licensee MDPI, Basel, Switzerland. This article is an open-access article distributed under the terms and conditions of the Creative Commons Attribution (CC BY) license [239].

Near-edge X-ray absorption fine structure spectroscopy (NEXAFS) was applied to analyze the local interactions between encapsulated substances and SWCNTs [145,146,147,148,149,150,151,152,153,154,155,156,157,158,159,160,161,162,163,164,165,166,167,168,169,170,171,172,173,174,175,176,177,178,179,180,181]. Figure 20 shows the NEXAFS spectra of the pristine and sulfur-containing sample without (SWCNT/S-1) and with sonication (SWCNT/S-2) before and after light illumination (SWCNT/S-1i, SWCNT/S-2i) [239].

**Figure 21:**
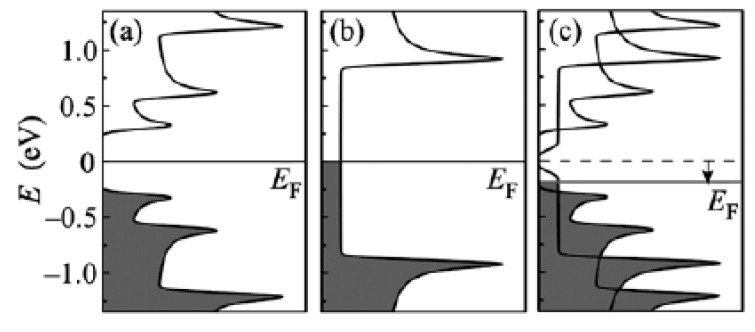
The schematic of band structures of pristine semiconducting (**a**) and metallic (**b**) SWCNTS and the Fermi level (E_F_) shift (shown with an arrow) in gallium selenide-filled SWCNTs (**c**). Reprinted from M.V. Kharlamova. Novel approach to tailoring the electronic properties of single-walled carbon nanotubes by the encapsulation of high-melting gallium selenide using a single-step process, JETP letters, V. 98, n 5, p. 272–277, 2013, Springer Nature [162].

The schematics of the modification of the electronic properties of filled SWCNTs are shown in Figure 21 [145,146,147,148,149,150,151,152,153,154,155,156,157,158,159,160,161,162,163,164,165,166,167,168,169,170,171,172,173,174,175,176,177,178,179,180,181]. 

## 6. Applications of Carbon Material and Chemically Functionalized Carbon Material in Electrochemical Devices

The electrochemical doping of materials can find applications [240,241,242,243,244] in supercapacitors [115,116], hydrogen storage [245,246], battery construction [247,248,249], sensors [240,244], and nanoelectronic devices [113,250,251,252]. For these experiments, special electrochemical cells, electrolytes, electrode materials, and other parameters are important [141]. Recent works include electrochemical studies of carbon materials [253,254,255,256,257,258,259,260,261,262,263,264,265,266,267,268,269,270,271,272,273,274,275,276,277,278,279,280,281].

**Figure 22:**
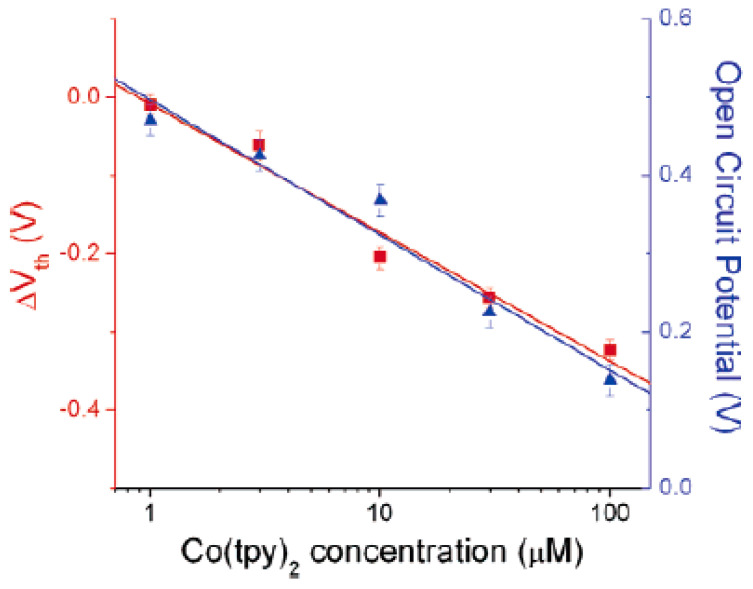
Threshold voltage shifts of SWCNT FET (red squares, left axis) and open-circuit potential between the working and reference electrode (blue triangles, right axis). Reprinted with permission from Larrimore L et al. Nano Lett. 6 1329 (2006). Copyright 2006 American Chemical Society [251].

In Ref. [251], SWCNT field effect transistors (FET) can be used to reveal changes in the chemical potential. The gate-voltage dependence of the nanotube conductance was measured. This is because of the interaction of the molecules with the electrode and their redox chemistry. Figure 22 shows the threshold voltage shifts of SWCNT FET (red squares, left axis) and the open-circuit potential between the working and reference electrode (blue triangles, right axis) [251]. 

**Figure 23:**
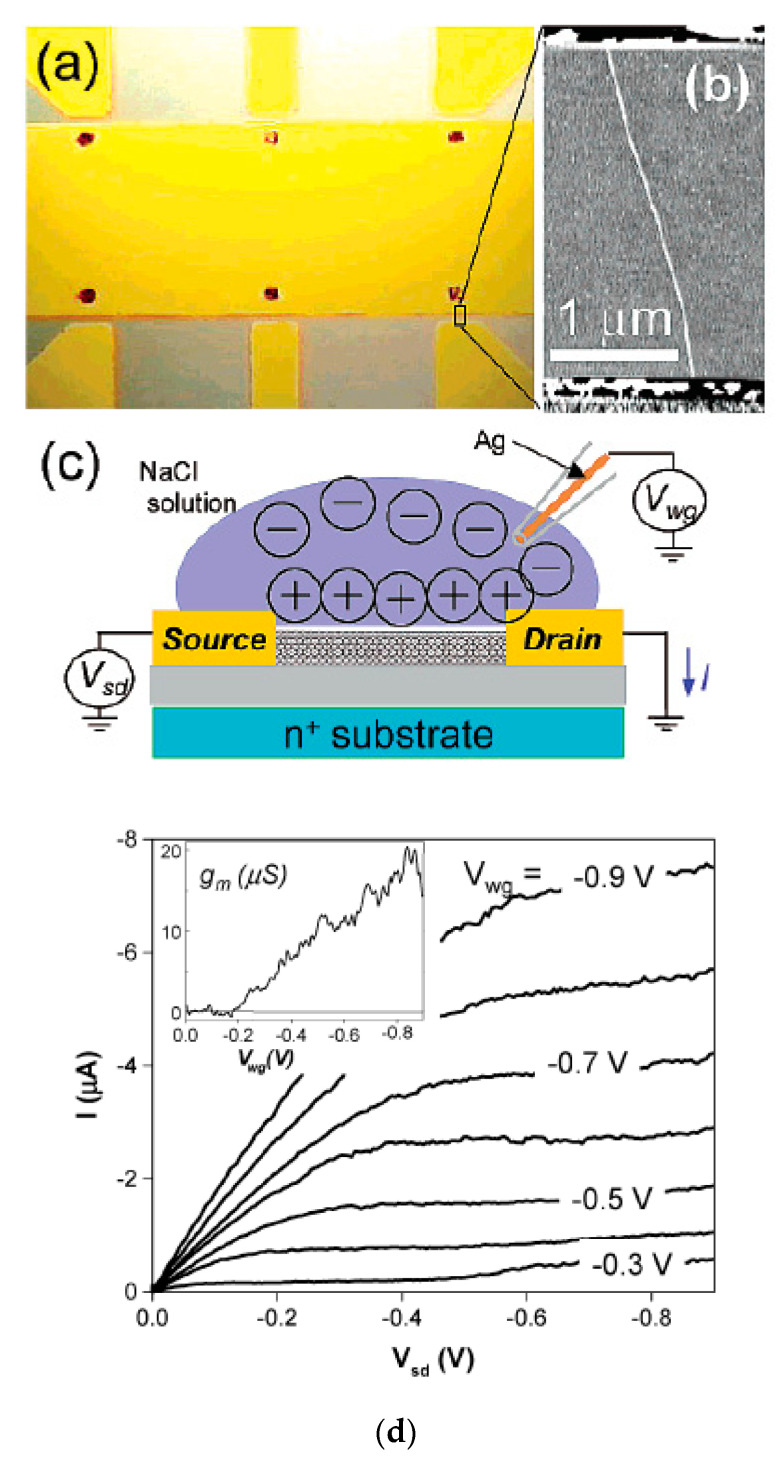
(**a**) Optical micrograph of the device. (**b**) Atomic force microscopy image of a tube between two electrodes. The tube diameter is 1.9 nm. (**c**) Schematic of the electrolyte gate measurement. (**d**) Current versus source drain voltage I-V_sd_ characteristics of the device. The inset shows the transconductance g_m_ = dI/dV_wg_ taken at V_sd_ = −0.8 V. Reprinted with permission from Rosenblatt S et al. Nano Lett. 2 869 (2002). Copyright 2002 American Chemical Society [252].

The authors of Ref. [252] made high perform FET-transistor form semiconducting SWCNTs with a diameter of 1.9 nm. Figure 23a shows an optical image of the transistor. They used six source electrodes. Figure 23b shows an atomic force microscopy (AFM) image of a tube in the FET. Figure 23c demonstrates the schematic of the electrolyte gate measurement [252]. The authors obtained very high device mobilities and transconductances. This makes semiconducting SWCNTs very useful for electronic applications and sensing (Figure 23d) [252].

**Figure 24:**
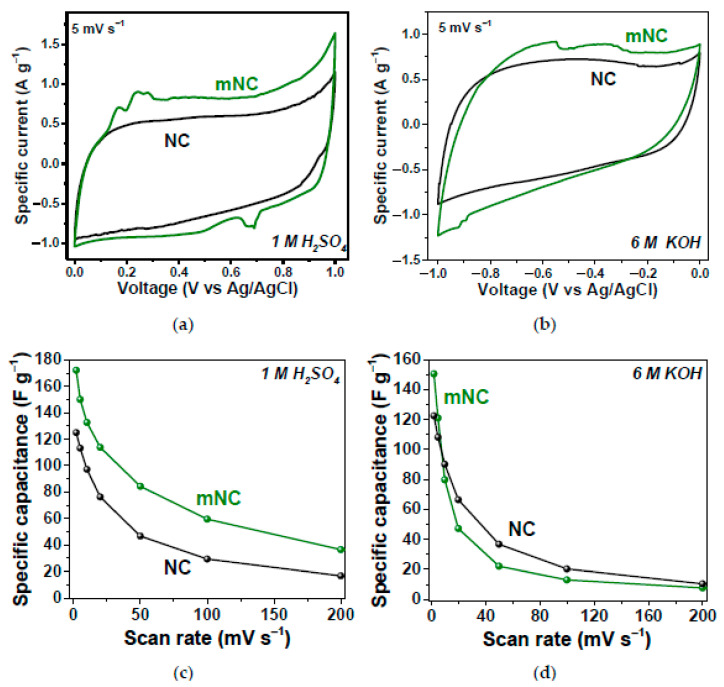
Specific current vs. voltage dependence of NC and mNC in 1 M H_2_SO_4_ (**a**) and 6 M KOH (**b**); specific capacitance vs. scan rate dependence of NC and mNC in 1 M H_2_SO_4_ (**c**) and 6 M KOH (**d**). Copyright 2022 by the author. Licensee MDPI, Basel, Switzerland. This article is an open-access article distributed under the terms and conditions of the Creative Commons Attribution (CC BY) license [268].

In Ref. [268], the nitrogen-doped carbon (NC) and modified-with-ammonia nitrogen-doped carbon (mNC) were tested as working electrodes of supercapacitors in three-electrode cells using 1 M H_2_SO_4_ and 6 M KOH electrolytes. Figure 24 shows the electrochemical performance of the samples, which show excellent characteristics [268]. 

**Figure 25:**
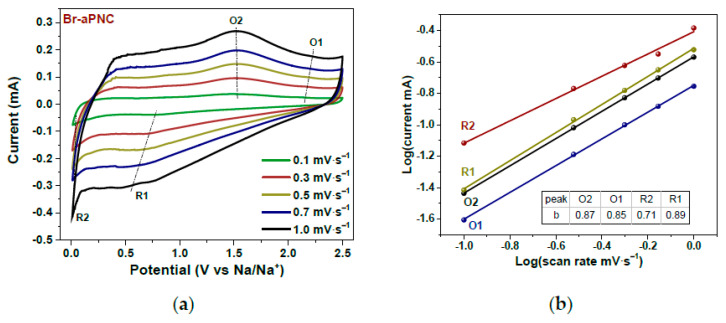
Current vs. potential curves of Br-aPNC (**a**) measured at scan rates of 0.1–1.0 mV s^−1^ and log(current)–log(scan rate) plots (**b**) plotted for oxidation (O) and reduction (R) peaks. Copyright 2022 by the author. Licensee MDPI, Basel, Switzerland. This article is an open-access article distributed under the terms and conditions of the Creative Commons Attribution (CC BY) license [262].

In Ref. [262], the kinetics of sodium storage were studied in brominated activated in alkali porous nitrogen-doped carbon. Figure 25 shows the current–potential curves of Br-aPNC (Figure 25a) measured at scan rates of 0.1–1.0 mV s^−1^ and the log(i)–log(v) plots (Figure 25b) obtained for oxidation and reduction peaks [262]. This behavior improves the storage and high-rate capability of carbon materials in sodium-ion batteries. 

**Figure 26:**
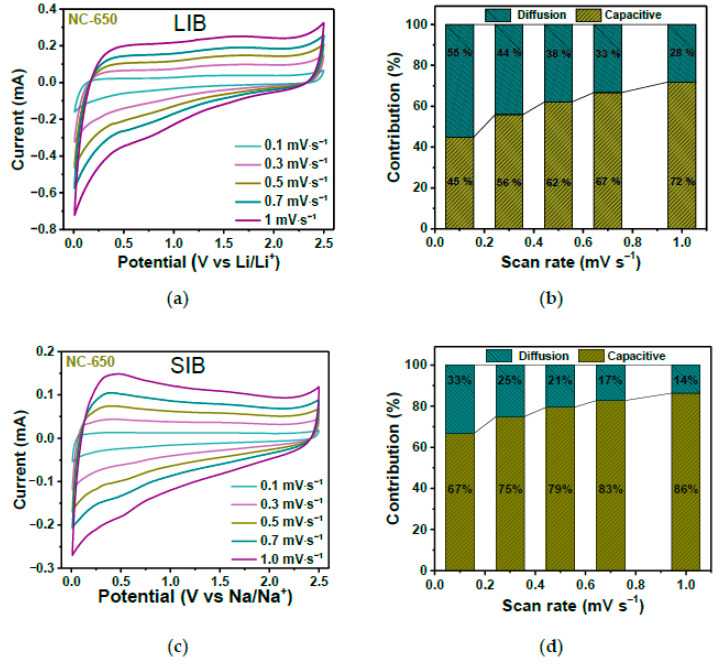
The current vs. potential measurements (**a**,**c**) and diffusion and pseudocapacitive contributions to the electrochemical storage at various scanning rates (**b**,**d**) in lithium-ion batteries and sodium-ion batteries. Copyright 2023 by the author. Licensee MDPI, Basel, Switzerland. This article is an open-access article distributed under the terms and conditions of the Creative Commons Attribution (CC BY) license [263].

In Ref. [263], annealed nitrogen-doped carbon showed an excellent performance in both lithium-ion batteries and sodium-ion batteries. Figure 26a,c shows the current vs. potential measurements at various scanning rates. The diffusion and pseudocapacitive contributions to the electrochemical storage for different scan rates are shown in Figure 26b,d. It is visible that by increasing the scan rate, the pseudocapacitive contribution increases [263]. 

**Figure 27:**
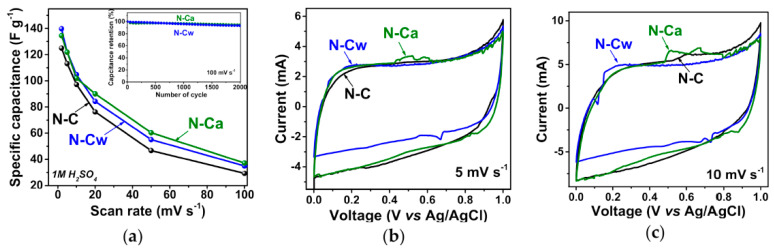
(**a**) Specific capacitances and current vs. voltage curves at scan rates of (**b**) 5 and (**c**) 10 mV s^−1^ of N-C, N-Cw, and N-Ca samples. The inset in (**a**) presents capacitance retention plots for N-C and N-Ca during 2000 cycles at 100 mV s^−1^. Copyright 2020 by the author. Licensee MDPI, Basel, Switzerland. This article is an open-access article distributed under the terms and conditions of the Creative Commons Attribution (CC BY) license [275].

In Ref. [275], the electrochemical properties of nitrogen-doped carbon material were investigated. Figure 27a shows the specific capacitance plotted vs. scan rate for pristine nitrogen-doped carbon (N-C) and that after hydrothermal treatment in water (N-Cw) and ammonia solution (N-Ca). Figure 27b,c show the current vs. voltage measurements at scan rates of 5 and 10 mV s^−1^ [275].

**Figure 28:**
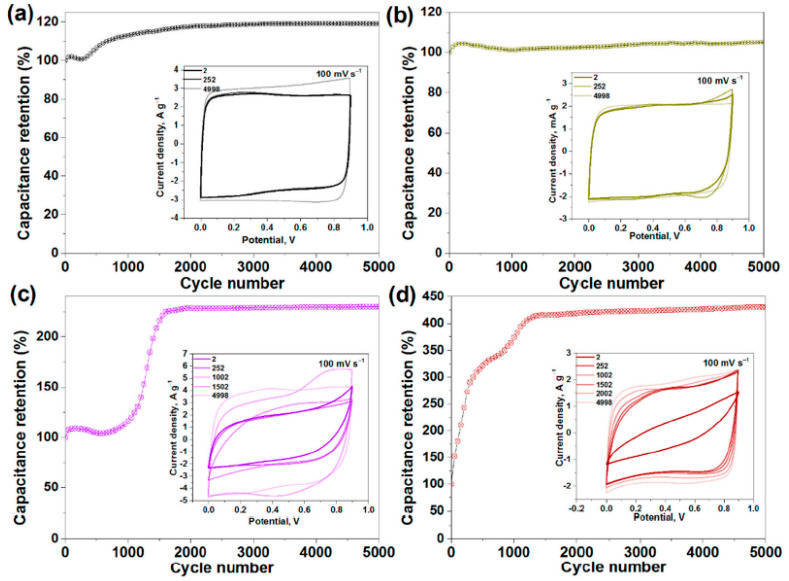
Capacitance retention versus cycle number measurements, with insets showing the current density versus potential measurements for pristine (SW) (**a**), split (SW_DC) (**b**), and fluorinated F-SW (**c**) and F-SW_DC (**d**) electrodes at a scan rate of 100 mV s^−1^. Copyright 2021 by the author. Licensee MDPI, Basel, Switzerland. This article is an open-access article distributed under the terms and conditions of the Creative Commons Attribution (CC BY) license [276].

In Ref. [276], the electrochemical properties of pristine, sonicated, and fluorinated SWCNTs were investigated. Figure 28 shows the current density vs. potential measurements for pristine (SW), split (SW_DC), and fluorinated F-SW, and the F-SW_DC electrodes at a scan rate of 100 mV s^−1^ show a nearly rectangular shape. Figure 28 also shows the capacitance retention vs. cycle number measurements [276]. 

**Figure 29:**
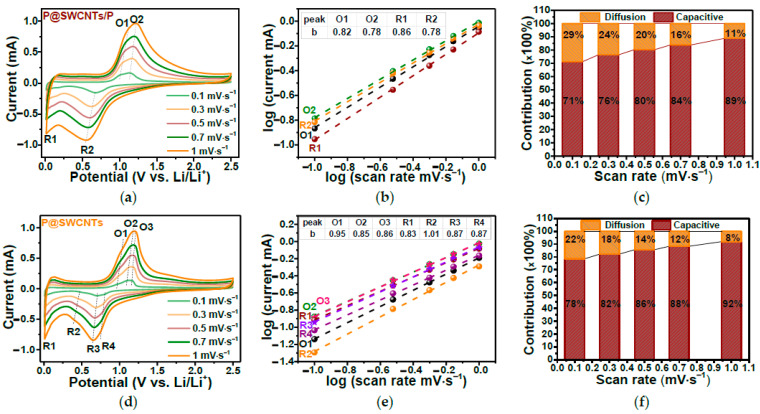
The current versus potential dependence (**a**,**d**), the log(current) vs. log(scan rate) (**b**,**e**), and the diffusion and capacitive contributions for different scan rates (**c**,**f**) of P-filled SWCNTs (P@SWCNT/P) and treated P@SWCNT. Copyright 2023 by the author. Licensee MDPI, Basel, Switzerland. This article is an open-access article distributed under the terms and conditions of the Creative Commons Attribution (CC BY) license [277].

In Ref. [277], the electrochemical properties of SWCNTs filled with red phosphorous were investigated. Figure 29a,d show the current vs. potential dependence of P-filled SWCNTs (P@SWCNT/P) and those treated by filtering, sonication, and drying samples (P@SWCNT). Figure 29b,e show the log(current) vs. log(scan rate). Figure 29c,f demonstrate the diffusion and capacitive contributions for different scan rates [277].

## 7. Conclusions

The filled nanotubes show great promise in many areas [13,253] and are set to advance functional materials of the future. For instance, carbon nanotubes have been used with metal oxides as cheap and stable nanocatalysts in the alcohol oxidation processes for fuel cells [282]. There are many filling materials [6,7,145,146,147,148,149,150,151,152,153,154,155,156,157,158,159,160,161,162,163,164,165,166,167,168,169,170,171,172,173,174,175,176,177,178,179,180,181] that can create devices with an appropriate efficiency, intensity, and color. They are not patented yet because their construction requires five to ten years. I think that the filling of single-walled carbon nanotubes is a very promising achievement; there are no other ways to create devices than to start from laboratory synthesis in a flask, and the applications in car lights are the most recent. The state corporations in Russia, Europe, the USA, and different countries are interested in developments [283,284,285,286,287,288,289,290,291,292,293,294,295,296,297,298,299,300,301,302].

## Data Availability

Data are available on request to the first author (M.V.K.).
